# Electroactive Biomaterials for Facilitating Bone Defect Repair under Pathological Conditions

**DOI:** 10.1002/advs.202204502

**Published:** 2022-12-01

**Authors:** Boon Chin Heng, Yunyang Bai, Xiaochan Li, Lee Wei Lim, Wang Li, Zigang Ge, Xuehui Zhang, Xuliang Deng

**Affiliations:** ^1^ Central Laboratory Peking University School and Hospital of Stomatology Beijing 100081 P. R. China; ^2^ School of Medical and Life Sciences Sunway University Darul Ehsan Selangor 47500 Malaysia; ^3^ Department of Geriatric Dentistry Peking University School and Hospital of Stomatology Beijing 100081 P. R. China; ^4^ Neuromodulation Laboratory School of Biomedical Sciences Li Ka Shing Faculty of Medicine The University of Hong Kong Pokfulam Hong Kong P. R. China; ^5^ Department of Biomedical Engineering Peking University Beijing 100871 P. R. China; ^6^ Department of Dental Materials & Dental Medical Devices Testing Center Peking University School and Hospital of Stomatology Beijing 100081 P. R. China; ^7^ National Engineering Research Center of Oral Biomaterials and Digital Medical Devices NMPA Key Laboratory for Dental Materials Beijing Laboratory of Biomedical Materials & Beijing Key Laboratory of Digital Stomatology Peking University School and Hospital of Stomatology Beijing 100081 P. R. China

**Keywords:** biomaterials, bone, degenerative, disease, electric, scaffold

## Abstract

Bone degeneration associated with various diseases is increasing due to rapid aging, sedentary lifestyles, and unhealthy diets. Living bone tissue has bioelectric properties critical to bone remodeling, and bone degeneration under various pathological conditions results in significant changes to these bioelectric properties. There is growing interest in utilizing biomimetic electroactive biomaterials that recapitulate the natural electrophysiological microenvironment of healthy bone tissue to promote bone repair. This review first summarizes the etiology of degenerative bone conditions associated with various diseases such as type II diabetes, osteoporosis, periodontitis, osteoarthritis, rheumatoid arthritis, osteomyelitis, and metastatic osteolysis. Next, the diverse array of natural and synthetic electroactive biomaterials with therapeutic potential are discussed. Putative mechanistic pathways by which electroactive biomaterials can mitigate bone degeneration are critically examined, including the enhancement of osteogenesis and angiogenesis, suppression of inflammation and osteoclastogenesis, as well as their anti‐bacterial effects. Finally, the limited research on utilization of electroactive biomaterials in the treatment of bone degeneration associated with the aforementioned diseases are examined. Previous studies have mostly focused on using electroactive biomaterials to treat bone traumatic injuries. It is hoped that this review will encourage more research efforts on the use of electroactive biomaterials for treating degenerative bone conditions.

## Graphical abstract

Various diseases such as type II diabetes, osteoporosis, periodontitis, osteoarthritis, rheumatoid arthritis, osteomyelitis, and metastatic osteolysis can lead to bone degenerative conditions. Electroactive scaffolds can promote bone regeneration under pathological conditions by recapitulating the natural electrophysiological microenvironment of healthy bone tissues. This is achieved through enhancement of osteogenesis and angiogenesis, suppression of inflammation and osteoclastogenesis, as well as anti‐bacterial effects.

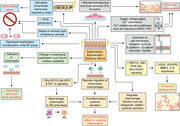

## 1 Introduction

Bone is a hard mineralized tissue composed predominantly of inorganic hydroxyapatite (Ca_10_ (PO_4_)_6_ (OH)_2_) nanocrystals interspersed with various organic components including cells, collagen fibers, and various other extracellular matrix molecules.^[^ ^1^ ^]^ In addition to providing structural support for the body, bones also protects soft and vulnerable tissues and organs, and serve as attachment points for tendons and muscles to facilitate physical movement.^[^ ^2^ ^]^

The number of orthopedic clinical cases involving deficient bone healing and regeneration associated with various diseases conditions has been increasing, mainly due to the rapidly aging population and sedentary lifestyles and unhealthy diets associated with urban living.^[^ ^3^ ^]^ Several common diseases including type II diabetes, periodontitis, osteoporosis, osteoarthritis, rheumatoid arthritis, osteomyelitis, and metastatic osteolysis can lead to bone degeneration and loss, or compromised bone healing and regeneration after injury.^[^ ^4^, ^5^, ^6^, ^7^, ^8^, ^9^ ^]^ Moreover, the prognosis of bone defect healing under such disease conditions is usually very poor,^[^ ^4^, ^5^, ^6^, ^7^, ^8^, ^9^ ^]^ and often requires therapeutic intervention.

In recent years, tissue engineering has offered a promising therapeutic strategy to facilitate bone repair and regeneration.^[^ ^10^ ^]^ Tissue engineering involves combining and synergizing cells, bioactive factors, and scaffold biomaterials to enhance bone healing and regeneration under adverse pathological conditions. This review focuses on the scaffold biomaterials, in particular electroactive scaffold biomaterials. Although scaffold biomaterials developed for bone repair and regeneration have often focused on more potent therapeutic strategies, for example the controlled release of bioactive factors such as small molecule drugs, peptides and large protein growth factors; these have certain disadvantages such as their limited active half‐life in vivo, as well as tendency to diffuse away from the target sites, which not only reduces their potency, but may also exert adverse side effects at ectopic sites. Focusing on the biophysical parameters of scaffold biomaterials such as their electroactive properties, do not incur such disadvantages.

Based on evidence that bone traumatic injuries (such as fracture) drastically alters the electrical potential of bone tissue;^[^ ^11^ ^]^ it is reasonable to hypothesize that various disease pathologies that lead to degenerative bone conditions involve degradation of the natural electrophysiological properties of bone tissue. Hence the utilization of electroactive biomaterials is a biomimetic approach to promote bone regeneration by restoring the natural bioelectrical properties of healthy bone tissue. Electroactive biomaterials that could realize electrodeless, wireless and self‐charging functions (such as piezoelectricity) would be much preferred for tissue engineering applications. These electroactive biomaterials can closely mimic and recapitulate the bioelectrical properties of healthy bone tissues,^[^ ^12^ ^]^ providing a favorable and conducive microenvironment to promote osteogenesis and bone regeneration under various pathological conditions.

Nevertheless to date, the overwhelming majority of pre‐clinical studies on the application of electroactive scaffold biomaterials in bone tissue engineering and orthopedic surgery, have been based mainly on young and healthy animal models, with particular focus on the treatment of bone traumatic injuries. Such data cannot be readily extrapolated to the human clinical model because many patients, especially the elderly, often suffer from various diseases that result in degenerative bone conditions,^[^ ^4^, ^5^, ^6^, ^7^, ^8^, ^9^ ^]^ thereby compromising bone healing and repair. Hence, newly‐developed biomaterials for bone repair and regeneration that work well in young and healthy animals, may not necessarily work well in elderly patients afflicted with various pathological conditions. Although some excellent reviews have already been published on application of electroactive biomaterials in bone tissue engineering and orthopedic surgery that have provided a broad perspective and critical in‐depth analysis;^[^ ^10^, ^12^, ^13^ ^]^ none of these have exclusively focused on bone repair and regeneration under disease conditions, which will therefore be the subject of this review.

## 2 Bone is an Electroactive, Electrosensitive, and Electroresponsive Tissue

**Figure 1:**
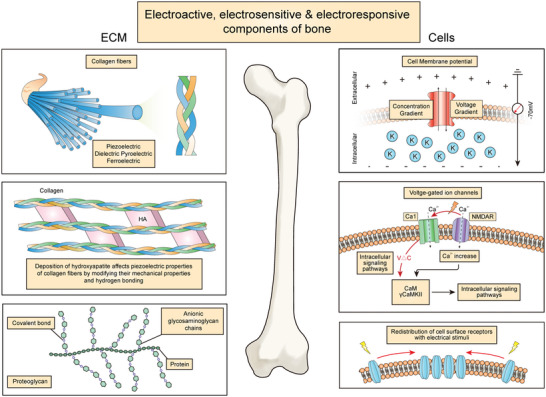
Bone is an electroactive, electrosensitive and electroresponsive tissue.

In designing and fabricating electroactive scaffolds for promoting bone repair and regeneration under various disease conditions, it is necessary to understand the bioelectrical properties of bone tissue that we are trying to mimic and recapitulate. This is best thought of as a combination and overlap of dielectric, piezoelectric, pyroelectric, and ferroelectric properties, together with electro‐osmosis and streaming potential (**Table** **1**),^[^ ^14^, ^15^, ^16^, ^17^, ^18^, ^19^ ^]^ which arise from the interactions between the various inorganic and organic components (**Figure** **1**), under biomechanical stimuli associated with daily physical activities. The dielectric property of bone tissue can be attributed to the separation of hydrogen bonds between hydroxyapatite (HA) and collagen, in the presence of an external electrical field.^[^ ^14^ ^]^ The piezoelectric property is attributed to collagen fibers sliding against each other upon application of mechanical force to the bone tissue during normal daily physical activity. This leads to the separation and polarization of charged groups on collagen molecules resulting in the formation of a dipole, thereby generating a piezoelectric potential.^[^ ^15^ ^]^ The pyroelectric property is thought to arise from the distortion of the collagen triple helical structure upon temperature change, resulting in polarization of charged amino acid residues that generates a pyroelectric potential.^[^ ^16^ ^]^ The ferroelectric property can be attributed to spontaneous changes in the polarization of collagen fibers, even in the absence of an external electric field.^[^ ^17^ ^]^ Electro‐osmosis is the flow of interstitial fluid through the canaliculi and lacunae of bone tissue, which is induced by piezoelectric potential generated through physical activity.^[^ ^18^ ^]^ The streaming potential is the electrical potential generated by the flow of ionic fluid through the pores within bone tissue (canaliculi and lacunae) during physical activity.^[^ ^19^ ^]^

**Table 1:** Summary of the bioelectrical properties of bone tissue

Electrical properties	Definition	Mechanisms	Key references
Dielectric properties	Capacity for polarization of negative and positive charges upon exposure to an electric field	Application of an electrical field leads to separation of hydrogen bonds between hydroxyapatite (HA) and collagen	Ray and Behari^[^ ^14^ ^]^
Piezoelectric properties	Capacity to generate electricity upon application of mechanical stimuli	Mechanical force causes sliding of collagen fibers against each other. This results in dipole rearrangement and subsequent separation and polarization of –CO– and –NH– groups on collagen, which in turn generates electrical potential	Nair et al.^[^ ^15^ ^]^
Pyroelectric properties	Capacity to generate electricity via polarization of negative and positive charges due to changing temperature	Changing temperature distorts collagen triple helical structure, resulting in polarization of charged amino acid residues, thereby generating electrical potential	Ravi et al.^[^ ^16^ ^]^
Ferroelectric properties	Capacity to display reversible spontaneous polarization and hysteresis loop even in the absence of an external electric field	In the absence of an external electric field, collagen fibers can spontaneously and reversibly change their orientation in different directions	El Messiery et al.^[^ ^17^ ^]^
Streaming potential	Electrical potential is generated by fluid and ion flow, driven by mechanical loading of bone	Mechanical stimuli on bone due to physical activity, drives the flow of fluids containing charged ions through the canaliculi and pores of bone tissue. An electric potential is generated by this flow of ions against the charged bone surface.	Gross and Williams^[^ ^18^ ^]^
Electro‐osmosis	Fluid flow through a narrow channel is driven by an electric field	Interstitial fluid flow through the channels and pores of bone tissue (canaliculi, lacunae) is driven by endogenous electrical potential in bone (e.g., piezoelectric potential)	Crolet et al.^[^ ^19^ ^]^

Collagen is the major organic component of bone tissue, making up 90% of the bone matrix. Collagen is a structural protein and is important for bone strength, as well as contributing significantly to the bioelectrical characteristics of bone tissue due to it's piezoelectric properties.^[^ ^15^ ^]^ Proteoglycans are another major organic component of bone tissue that also contribute to the bioelectrical properties. Proteoglycans consist of a protein core attached to glycosaminoglycan chains that are highly negatively charged, which in turn enable the sequestration of Ca^2+^ ions and various growth factors, and which contribute to the overall negative charge of the bone tissue. Hydroxyapatite is the major inorganic component of bone tissue that also influences the bioelectrical properties by restricting the accessibility of water molecules to form hydrogen bonds with collagen,^[^ ^20^ ^]^ while its high rigidity dampens the mechanical response of collagen fibers to tensional or compressive forces, thereby limiting the generation of piezoelectric stimuli.^[^ ^21^ ^]^

The three major cell lineages within bone tissue, osteoblasts, osteocytes, and osteoclasts, are also known to be electrosensitive and electroresponsive. For example, the transmembrane potential of these cells are altered upon response to either mechanical or electrical stimuli via mechanoresponsive and voltage‐gated ion channels, respectively. This in turn has wide‐ranging effects on cellular metabolism and various biological processes, leading to profound effects on bone tissue homeostasis and remodeling.^[^ ^22^ ^]^ Various signaling pathways in osteoblasts, osteocytes, and osteoclasts, in particular the calcineurin‐calmodulin‐NFAT signaling cascade and the extracellular signal‐related protein kinase (ERK) signaling pathway,^[^ ^23^, ^24^ ^]^ are highly sensitive to changes in intracellular Ca^2+^ levels modulated by voltage‐sensitive calcium channels in response to piezoelectric stimuli generated by the musculoskeletal system. Additionally, electrical stimuli have been shown to trigger the redistribution of various cell surface receptors such as fibronectin, epidermal growth factor (EGF), and concanavalin, which regulate cell migration, adhesion, and spreading.^[^ ^25^ ^]^

At present, to the best of our knowledge, there have not yet been any study that investigated how the electrophysiological properties of bone tissues are altered by various diseases that lead to degenerative bone conditions.^[^ ^4^, ^5^, ^6^, ^7^, ^8^, ^9^ ^]^ The only data of some relevance comes from the study of Zigman et al.^[^ ^11^ ^]^ that demonstrated drastic alteration of electrical potential upon bone fracture, which is subsequently restored to normal levels after fracture healing. It is thus reasonable to hypothesize that deficient homeostasis, remodeling, and regeneration of bone tissue associated with various diseases may be partly caused by aberrations in its bioelectrical properties arising from pathological conditions.

## 3 Bone Tissue Degeneration and Loss under Various Disease Conditions

### 3.1 Pathological Characteristics of Bone Repair under Disease Conditions and its Differences with Bone Defect Healing under Healthy Conditions

**Figure 2:**
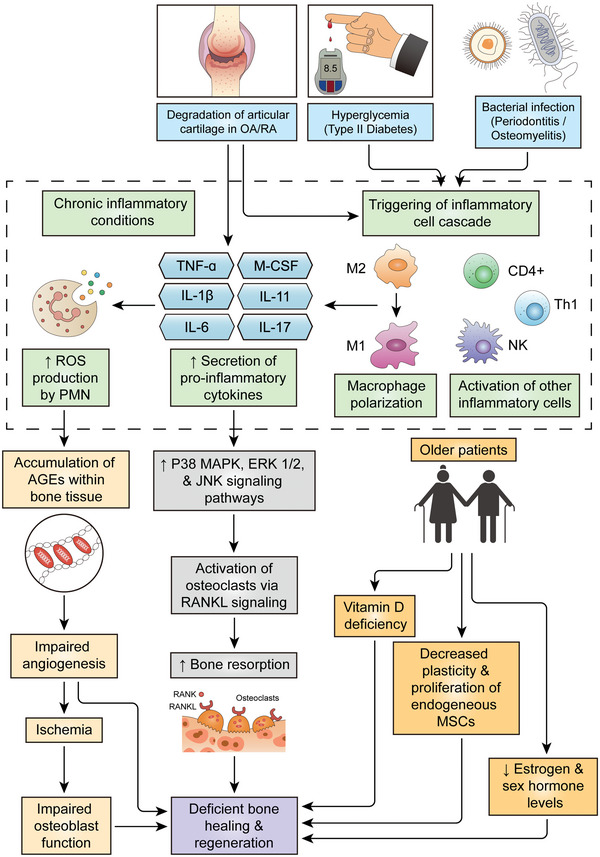
Similarities in causative mechanisms of deficient bone regeneration under various disease conditions.

Various disease conditions (summarized in **Table** **2**) often leads to deficient bone healing and regeneration, as compared to bone defect repair under healthy conditions. Although there is much variation in the pathological processes of different disease models that leads to degenerative bone conditions, the underlying causative mechanisms of deficient bone regeneration share a number of commonalities (**Figure** **2**). The most obvious of these is the presence of chronic inflammatory conditions, which may be provoked by bacterial infection, as in the case of periodontitis (Section 3.4) and osteomyelitis (Section 3.7), or may be induced by other factors such as hyperglycemic conditions in type II diabetes (Section 3.2), which promote polarization of macrophages to the pro‐inflammatory M1 phenotype. Otherwise, inflammation maybe an inherent hallmark of the disease itself, as in the case of osteoarthritis (Section 2.5) and rheumatoid arthritis (Section 2.6).

**Table 2:** Mechanisms of bone degeneration and loss under various disease conditions

Disease	Mechanisms of bone degeneration and loss	Key references
Type II Diabetes	Increased inflammation	Shen et al.^[^ ^26^ ^]^
	Increased ROS production/oxidative stress	Tang et al.^[^ ^27^ ^]^
	Accumulation of advanced glycation end products (AGEs)	Yamamoto & Sugimoto^[^ ^28^ ^]^ Karim & Bouxsein^[^ ^29^ ^]^
	Inhibition of angiogenesis	Lim et al.^[^ ^30^ ^]^ Caliaperoumal et al.^[^ ^31^ ^]^
	Impairment of osteoblast function	Peng et al.^[^ ^32^ ^]^ Wongdee et al.^[^ ^33^ ^]^ Park et al.^[^ ^34^ ^]^ Lee et al.^[^ ^35^ ^]^
Osteoporosis	Age‐related decline in hormonal/estrogen levels	Cheng et al.^[^ ^39^ ^]^ Gosset et al.^[^ ^40^ ^]^ Emmanuelle et al.^[^ ^41^ ^]^ Bjørnerem et al.^[^ ^42^ ^]^
	Age‐related decline in proliferative capacity, plasticity and number of endogenous mesenchymal stem cells	Li et al.^[^ ^43^ ^]^ Li et al.^[^ ^44^ ^]^
	Vitamin D deficiency in older patients	Al‐Daghri et al.^[^ ^45^ ^]^ Föger‐Samwald et al.^[^ ^46^ ^]^
Periodontitis	Inflammation triggered by bacterial infection/LPS on bacterial cell membrane	Page et al.^[^ ^50^ ^]^
	Inflammatory cascade promotes osteoclastogenesis/bone resorption	Zhou et al.^[^ ^53^ ^]^ Yang et al.^[^ ^54^ ^]^ Choi et al.^[^ ^55^ ^]^
	Inflammatory cascade suppresses osteoblast differentiation and function	Kaneshiro et al.^[^ ^56^ ^]^ Zou & Bar‐Shavit^[^ ^57^ ^]^
Osteoarthritis	Exposure of sub‐chondral bone to pro‐inflammatory cytokines secreted by OA chondrocytes	Chien et al.^[^ ^75^ ^]^
	Pro‐inflammatory cytokines promote osteoclastogenesis and bone resorption	Lee et al.^[^ ^76^ ^]^ Yang et al.^[^ ^77^ ^]^ Lam et al.^[^ ^78^ ^]^ Marahleh et al.^[^ ^79^ ^]^ Kudo et al.^[^ ^80^ ^]^
Rheumatoid arthritis	Inflammatory cascade promotes osteoclastogenesis and bone resorption	Goldring^[^ ^81^ ^]^
	Reduced bone mineralization due to deficiency of vitamin D, calcium and other mineral absorption	Bellan et al.^[^ ^82^ ^]^
Osteomyelosis	Bacterial infection causes apoptosis of osteoblasts, which promotes osteoclastogenesis	Claro et al.^[^ ^86^ ^]^
	Staphylococcal Protein A (SpA) secreted by *S. aureus* promotes osteoclastogenesis	Widaa et al.^[^ ^87^ ^]^ Chen et al.^[^ ^88^ ^]^ Mendoza Bertelli et al.^[^ ^89^ ^]^
	Inflammatory cascade activated by bacterial infection promote osteoclastogenesis and bone resorption	Lan et al.^[^ ^92^ ^]^
	Demineralization caused by increased expression of matrix metalloproteinase (perilacunar remodeling/osteocytic osteolysis)	Ormsby et al.^[^ ^93^ ^]^ Gunn et al.^[^ ^94^ ^]^
Metastatic osteolysis	Metastatic cells secrete growth factors and cytokines that promote osteoclastogenesis and bone resorption	Giannoni et al.^[^ ^99^ ^]^ Sethi et al.^[^ ^100^ ^]^ Lu et al.^[^ ^101^ ^]^ Andrade et al.^[^ ^102^ ^]^
	Altered metabolism and bioenergetics by metastatic cancer cells promote osteoclastogenesis and bone resorption	Tiedemann et al.^[^ ^104^ ^]^
	Metastatic cancer cells induce hypoxia and trigger HIF signaling that suppress osteoblastic differentiation and promote osteoclastogenesis	Xu et al.^[^ ^105^ ^]^ Todd and Johnson^[^ ^106^ ^]^

Elevated levels of inflammatory cytokines, in turn promotes increased production of reactive oxygen species (ROS), which originally evolved as a defensive mechanism against microbial infection, as in the case of periodontitis (Section 3.4) and osteomyelitis (Section 3.7). The increased ROS levels result in accumulation of advanced glycation end productions (AGEs) within bone tissue, which impairs angiogenesis and osteoblast function, thereby compromising bone repair and regeneration (Section 3.2). Yet another commonality among the various disease conditions, is a shift in the delicate balance between osteogenesis and osteoclastogenesis, in favour of the latter, which can be induced by elevated levels of inflammatory cytokines and ROS (Sections 3.4 and 3.7). Among older patients, deficient bone repair among the various disease models (Table 2), maybe further exacerbated by age‐related decline in the plasticity and proliferative capacity of endogenous bone marrow‐derived mesenchymal stem cells (Section 3.3). Additionally, vitamin D deficiency and decreased sex hormone levels in elderly people are also key factors that exacerbate deficient bone repair and regeneration, particularly in the case of osteoporosis (Section 3.3).

### 3.2 Type II Diabetes

Type II diabetes is associated with impaired bone healing and regeneration. There are several mechanisms contributing to deficient bone healing and regeneration, including increased production of reactive oxygen species (ROS) and proinflammatory mediators,^[^ ^26^, ^27^ ^]^ which in turn lead to increased accumulation of advanced glycation end products (AGEs),^[^ ^28^, ^29^ ^]^ inhibition of angiogenesis,^[^ ^30^, ^31^ ^]^ and impairment of osteoblast function.^[^ ^32^, ^33^, ^34^, ^35^ ^]^

In type II diabetes, bone healing and regeneration is often abnormally stagnated at the early proinflammatory stage of the healing process, which is characterized by upregulated expression of various proinflammatory cytokines and chronic inflammation.^[^ ^26^, ^27^ ^]^ Hyperglycemia associated with type II diabetes is known to trigger the production of chemokines that enhance the polarization of macrophages to the proinflammatory M1 state, while inhibiting their transition to the pro‐healing M2 phenotype, thereby impairing tissue regeneration and promoting inflammation.^[^ ^26^, ^27^ ^]^ Chronic inflammatory condition also induces the generation of ROS, leading to the accumulation of AGEs and impairment of bone healing and regeneration.^[^ ^28^, ^29^ ^]^ These processes fuel the “vicious circle” by promoting the upregulation of inflammatory cytokines in microenvironment of the local bone defect.

The overproduction and accumulation of AGEs in vascular tissues inhibits angiogenesis at the bone defect site, resulting in the altered function of endothelial cells, smooth muscle cells, and macrophages, which lead to complications such as micro‐ and macroangiopathy.^[^ ^30^, ^31^ ^]^ The subsequent ischemia creates an unfavorable environment for osteogenesis. Moreover the lowered oxygen tension under deficient angiogenesis impairs cross talk between bone vasculature and osteogenic precursor cells, resulting in reduced activation, recruitment, and differentiation of osteogenic precursor cells, which leads to increased bone porosity.^[^ ^32^, ^33^ ^]^

The accumulation of AGEs within bone tissues has also been reported to slow bone turnover by inhibiting osteoclastic and osteoblastic differentiation and activity, leading to lower bone quality and increased bone fragility.^[^ ^34^, ^35^ ^]^ Additionally, AGEs can alter the structural conformation by which collagen physically links to hydroxyapatite, which reduces the capacity of bone to dissipate mechanical energy and to deform under mechanical stress, and results in increased susceptibility to fractures at low levels of mechanical strain.^[^ ^36^ ^]^ The altered bone mechanical properties due to AGEs and the increased bone porosity due to deficient angiogenesis are two key factors that contribute to increased bone fragility in patients with type II diabetes.

### 3.3 Osteoporosis

Osteoporosis is a systemic bone disease that is characterized by microarchitectural deterioration of bone tissue, resulting in progressive loss of bone mass that increases bone fragility and vulnerability to fractures.^[^ ^37^ ^]^ It increases with age and is particularly common among older post‐menopausal women.^[^ ^37^ ^]^ Osteoporosis can be attributed to increasing imbalance between bone formation and bone resorption, in which bone resorption exceeds bone formation. Bone homeostasis involves continual dynamic tissue remodeling via osteoclast‐mediated resorption and osteoblast‐mediated formation of new bone tissue.^[^ ^38^ ^]^ The regulation of bone homeostasis is a delicate balance between bone formation and bone resorption, which is critical for ensuring adequate bone density and mechanical strength. There are currently three main proposed underlying causative mechanisms of osteoporosis: i) age‐related decline in hormonal levels, particularly estrogen deficiency in older post‐menopausal women;^[^ ^39^, ^40^, ^41^, ^42^ ^]^ ii) age‐related decrease in the proliferative potential, plasticity, and number of endogenous mesenchymal stem cells;^[^ ^43^, ^44^ ^]^ and iii) vitamin D deficiency in older patients.^[^ ^45^, ^46^ ^]^

Estrogen, testosterone, and parathyroid hormone (PTH) play key roles in bone tissue remodeling by promoting bone formation and inhibiting bone resorption.^[^ ^39^ ^]^ In older women, there is a sharp decline in estrogen production after menopause, which leads to a substantial decline in bone mineral density (BMD).^[^ ^40^ ^]^ In contrast, older men show a more gradual decrease in BMD in tandem with a steady decline in hormonal levels.^[^ ^41^ ^]^ In both aging men and women, increasing levels of sex hormone‐binding globulin is thought to inactivate both estrogen and testosterone, resulting in progressive BMD decline.^[^ ^42^ ^]^ By the age of 60, age‐related hormonal changes result in equal rates of bone loss in both men and women accompanied by increased risk of osteoporosis.^[^ ^42^ ^]^

The number of endogenous mesenchymal stem cells also decreases with age, accompanied by an impairment in their ability to differentiate into osteoblasts.^[^ ^43^, ^44^ ^]^ It was reported that with aging, there is an increased propensity for bone marrow‐derived mesenchymal stem cells to undergo adipogenic rather than osteogenic differentiation, resulting in the increased accumulation of fat in the bone marrow of older patients.^[^ ^43^ ^]^ The decrease in the number and function of osteoblasts with age leads to an imbalance in new bone formation versus bone resorption, and is a primary cause of age‐related osteoporosis. The accumulation of adipocytes in the bone marrow can also promote apoptosis of osteoblasts.^[^ ^44^ ^]^

Another causative mechanism of increased bone resorption with aging is vitamin D deficiency in older patients.^[^ ^45^, ^46^ ^]^ The resulting secondary hyperparathyroidism caused by vitamin D deficiency has been reported to increase bone resorption via osteoclast activation.^[^ ^45^, ^46^ ^]^

It is currently believed that impairment of bone formation rather than increase in bone resorption, plays a more important role in the pathology of age‐related osteoporosis, which primarily arises from decreased number and activity of bone forming cells due to lower hormonal levels with increasing age.

### 3.4 Periodontitis

Periodontitis is a chronic inflammatory disease characterized by gradual destruction and loss of alveolar bone, eventually leading to tooth loss due to prolonged and persistent activation of osteoclasts within the periodontium.^[^ ^47^, ^48^ ^]^ It is caused by the formation of subgingival bacterial plaque biofilms composed mainly of gram‐negative bacteria, in particular *Porphyromonas gingivalis* (*P. gingivalis*), *Eschericia coli* (*E. coli*), and *Aggregatibacter actinomycetemcomitans* (*A. actinomycetemcomitans*).^[^ ^49^ ^]^ These bacteria contain lipopolysaccharide (LPS) within their outer membrane that can act as a virulence factor and endotoxin, which induces tissue dysfunction and provokes an inflammatory response,^[^ ^50^ ^]^ as well as directly stimulate osteoclastogenesis.^[^ ^51^ ^]^ The innate and acquired host immunity is impaired due to persistent infection by periodontopathogenic bacteria, inevitably resulting in bone tissue degeneration.

The activation of the inflammatory cascade in periodontitis also leads to alveolar bone loss due to the action of inflammatory cytokines on osteoblasts and receptor activator of nuclear factor‐B ligand (RANKL)‐expressing hematopoietic cells, resulting in osteoclast differentiation and activation.^[^ ^52^ ^]^ In response to periopathogenic bacteria, various cell types in the local microenvironment synthesize a diverse array of proinflammatory cytokines such as tumor necrosis factor (TNF), interleukin‐1beta (IL‐1*β*), interleukin‐6 (IL‐6), and interleukin‐17 (IL‐7) that upregulate RANKL production and/or exert synergistic effects on RANKL signaling, which in turn accelerates osteoclast‐mediated bone resorption.^[^ ^53^, ^54^, ^55^ ^]^ Furthermore, IL‐1*β*, IL‐6, and TNF also have potent anti‐osteoblastic activity that suppress osteoblast differentiation and function.^[^ ^56^, ^57^ ^]^

Additionally, LPS directly stimulates osteoclastogenesis by acting on Toll‐like receptor 4 (TLR4) expressed by osteoclast progenitor cells and by osteoblasts involved in osteoclastogenesis.^[^ ^53^ ^]^ Previous studies have reported that LPS can stimulate the expression of prostaglandin E2 (PGE2) and RANKL via interaction with TLR4 expressed on osteoblasts,^[^ ^54^ ^]^ as well as promote osteoclast formation when bone marrow cells (including osteoclast progenitor cells) are co‐cultured with osteoblasts.^[^ ^55^ ^]^ Nevertheless, it was reported that LPS alone is unable to promote the formation of osteoclasts from precursor cells, which was found to require pre‐treatment with RANKL.^[^ ^57^ ^]^ The direct administration of LPS to periodontal tissues of animal models led to the resorption of alveolar bone by osteoclasts, which is similar to periodontitis.^[^ ^58^, ^59^ ^]^

Besides inflammatory cytokines and LPS, elevated ROS level is another major factor causing alveolar bone loss in periodontitis.^[^ ^60^ ^]^ Polymorphonuclear leukocytes (PMN) involved in the immune response to periopathogenic bacteria synthesize ROS to counter the invading pathogens.^[^ ^61^ ^]^ Immune cell‐secreted interleukin‐4 activating nicotinamide adenine dinucleotide phosphate (NADPH) oxidases (NOX) can generate ROS in response to bacterial LPS.^[^ ^62^ ^]^ The elevation of ROS level favors osteoclastogenesis over osteoblastogenesis via the activation of various signaling pathways including mitogen‐activated protein kinases (MAPKs), such as extracellular signal‐regulated kinases (ERK1/2), c‐Jun‐N terminal kinase (JNK), and p38 MAPK.^[^ ^63^, ^64^, ^65^ ^]^ Additionally, ROS can directly degrade the extracellular matrix in bone tissue,^[^ ^66^ ^]^ further contributing to alveolar bone loss.

### 3.5 Osteoarthritis

Osteoarthritis (OA) is a degenerative joint disease caused by mechanical wear and tear. It predominantly involves the destruction of articular cartilage, although there is also a gradual degeneration and loss of the subchondral bone that lies adjacent to cartilage tissue and provides it with nutritional and mechanical support.^[^ ^67^, ^68^ ^]^ The role of subchondral bone in OA pathology has gained more attention in recent years, with the gradual realization that the pathophysiology of OA involves intimate cross talk between subchondral bone and articular cartilage.^[^ ^69^, ^70^ ^]^ Indeed, a functional complex known as the bone–cartilage unit is formed by subchondral bone and cartilage, which is implicated in OA pathophysiology at the mechanical and biochemical levels.^[^ ^69^, ^70^ ^]^ It was reported that asymptomatic subchondral bone marrow lesions appear earlier than cartilage destruction or OA pain.^[^ ^71^, ^72^ ^]^

Notably, there is alteration of bone remodeling rates during OA progression due to spontaneous activation or inactivation of bone resorption by osteoclasts.^[^ ^73^ ^]^ As a result, activation of bone resorption may also be evident in the subchondral bone microenvironment in the early stages of OA. During early‐stage OA, excessive mechanical loads on adjacent subchondral bone are reduced due to self‐repair of articular cartilage. As a result of this underloading, the ratio of the expression of receptor activator of nuclear factor *κ*B ligand (RANKL)/osteoprotegerin (OPG) in osteocytes is increased, thereby leading to enhanced bone resorption activity due to excessive osteoclastogenesis.^[^ ^74^ ^]^

As OA progresses, subchondral bone is exposed to pro‐inflammatory cytokines secreted by OA chondrocytes.^[^ ^75^ ^]^ There is upregulation of IL‐1*β* in primary chondrocytes due to alteration of joint biomechanical properties,^[^ ^75^ ^]^ which in turn induce osteoclast formation via upregulation of RANKL expression by osteoblasts.^[^ ^76^ ^]^ Additionally, OA chondrocytes excessively secrete tumour necrosis factor (TNF)‐*α* and IL‐6.^[^ ^77^ ^]^ TNF‐*α* can directly promote osteoclast differentiation via activation of c‐Jun NH2‐terminal protein kinase (JNK) and NF‐*κ*B in a RANKL‐independent manner;^[^ ^78^ ^]^ as well as by stimulation of RANKL expression in osteocytes.^[^ ^79^ ^]^ IL‐6 can induce formation of tartrate‐resistant acid phosphatase (TRAP) and calcitonin receptor–positive osteoclasts from CD14‐positive peripheral blood mononuclear cells, in a RANKL‐independent manner via activation of the signal transduction factor gp130.^[^ ^80^ ^]^

### 3.6 Rheumatoid Arthritis

Rheumatoid arthritis (RA) is an autoimmune disease that results in joint degeneration. Similar to OA, RA also involves the activation of inflammatory pathways leading to degeneration and loss of subchondral bone tissue.^[^ ^81^ ^]^ Various pro‐inflammatory cytokines are produced by the inflamed RA synovium, which in turn promote osteoclastogenesis in the subchondral bone microenvironment. These include M‐CSF, IL‐1*β*, IL‐6, IL‐17, IL‐11, TNF‐*α*, and the parathyroid hormone‐related peptide, all of which promote osteoclast differentiation.^[^ ^81^ ^]^ The resulting formation of aggregates of lymphocytes and inflammatory macrophages within the bone marrow leads to local bone loss (osteopenia).^[^ ^81^ ^]^ Additionally, there is reduced bone mineralization in RA due to deficiencies of vitamin D, calcium, and other mineral absorption required for maintenance of healthy bones, which could lead to further bone degeneration.^[^ ^82^ ^]^

### 3.7 Osteomyelitis

Osteomyelitis is an inflammatory disease of the bone and bone marrow due to bacterial infection, most commonly *Staphylococcus aureus* (*S. aureus*), from hematogenous or traumatic sources.^[^ ^83^ ^]^ Diabetics and young children are particularly susceptible to developing this condition.^[^ ^83^ ^]^ Hematogenous bacterial infections are the most common cause of osteomyelitis and usually occur in children below 16 years old.^[^ ^84^ ^]^ Additionally, traumatic injury such as skin lesions, bone fractures, or surgical procedures can also be a route for bacterial infection into the wound, and invasion into the proximal bone tissue leads to osteomyelitis. Indeed, orthopedic surgery carries the risk of osteomyelitis via the transmission of bacteria from the skin to the surgery site.^[^ ^85^ ^]^

Bacterial infection of the bone tissue can cause apoptosis of osteoblasts, which in turn promotes osteoclast activity via upregulation of RANKL expression by osteoblasts, osteocytes, and polymorphonuclear leukocytes within tissues surrounding the infection site.^[^ ^86^ ^]^ Staphylococcal protein A (SpA) secreted by *S. aureus* has also been reported to upregulate RANKL expression by osteoblasts and polymorphonuclear leukocytes upon interaction with TNFR1 and TLR2.^[^ ^87^, ^88^ ^]^ Additionally, SpA can also activate TNF and EGF receptors on osteoclasts, which in turn upregulates the bone resorption capacity of these cells,^[^ ^89^ ^]^ leading to inhibited lacunae formation and bone necrosis, as observed in biopsies of human osteomyelitis patients and in animal models of osteomyelitis.^[^ ^90^, ^91^ ^]^ Moreover, increased secretion of various inflammatory cytokines at the infection site (e.g., IL‐8 secreted by osteoclasts, and IL‐6, TNF‐*α*, and IL‐1b secreted by immune cells and osteoblasts) can also promote osteoclastogenesis and bone resorption.^[^ ^92^ ^]^ Besides osteoclast‐related bone resorption, osteocytes are also known to reversibly remove bone minerals and remodel the organic phase of the bone matrix via the expression of matrix metalloproteinase, a process known as perilacunar remodeling or osteocytic osteolysis.^[^ ^93^ ^]^ It was reported that human osteocytes infected by *S. aureus* display increased expression of matrix metalloproteinase, suggesting that *S. aureus* infection promotes osteocytic osteolysis, which might contribute to osteomyelitis.^[^ ^94^ ^]^

### 3.8 Metastatic Osteolysis

Metastasis is the process by which cancer cells spread from one tissue/organ to another, and involves invasion and dissemination through the blood and lymph vessels. Typically, bone is the target organ for many solid tumor metastases, including prostate, breast, and lung carcinomas.^[^ ^95^ ^]^ The unique structure and microenvironment of the bone marrow, such as slow blood flow and the presence of various chemokines and growth factors, make it conducive for the growth of metastatic cancer cells,^[^ ^96^ ^]^ which then develop into metastatic lesions that destroy the bone tissue structure. The interaction of invading cancer cells with normal cells alters their cellular function, as well as the primary microenvironment of bone tissue.^[^ ^97^ ^]^ This can lead to bone tissue degeneration through the activation of osteoclasts and suppression of osteoblasts.^[^ ^98^ ^]^ Metastatic cancer cells can secrete IL‐11, TNF‐*α*, and various other factors that upregulate RANKL expression on osteoblasts, which in turn accelerate osteoclast formation and maturity.^[^ ^99^ ^]^ Metastatic cancer cells also express Notch ligands, Jagged1, and VCAM‐1, which are known to promote the activation of pre‐osteoclasts.^[^ ^100^, ^101^ ^]^ Additionally, metastatic cancer cells secrete macrophage‐stimulating protein (MSP) that can directly activate osteoclasts via the RON tyrosine kinase receptor.^[^ ^102^ ^]^

The increased osteoclastic activity induced by bone metastasis facilitates the degradation and release of various growth factors from the bone matrix, such as transforming growth factor beta (TGF‐*β*), insulin‐like growth factors (IGFs), fibroblast growth factors (FGFs), platelet‐derived growth factors (PDGFs), and bone morphogenetic proteins (BMPs), which in turn promote the proliferation of cancer cells, forming a “viscous circle” that drives the development of metastatic bone lesions.^[^ ^103^ ^]^ Additionally, cancer cells can alter their own bioenergetics for survival and function, such as adapting to glycolysis.^[^ ^104^ ^]^ Such a shift in energy metabolism by metastatic cancer cells within the bone alters the levels of cell‐permeable metabolites such as glucose, lactate, and pyruvate, which in turn make the bone metastasis microenvironment conducive for pathological osteolysis by energy‐expensive osteoclast resorption.

Another mechanistic pathway by which bone metastatic lesions promote increased osteoclastic activity is through hypoxia and hypoxia inducible factor (HIF) signaling. Hypoxia is a prominent characteristic of solid tumors, and has been demonstrated to inhibit the osteoblastic differentiation of mesenchymal stem cells,^[^ ^105^ ^]^ while enhancing the production of pro‐osteoclastic factors such as LOX via HIF‐signaling.^[^ ^106^ ^]^ LOX is known to activate osteoclastogenesis by promoting nuclear translocation of NFATc1, which is the master regulator of osteoclastogenesis.^[^ ^106^ ^]^

## 4 Mechanisms by which Electroactive Scaffolds Promote Bone Regeneration and Healing under Various Disease Conditions

### 4.1 Enhancement of Osteogenesis

**Figure 3:**
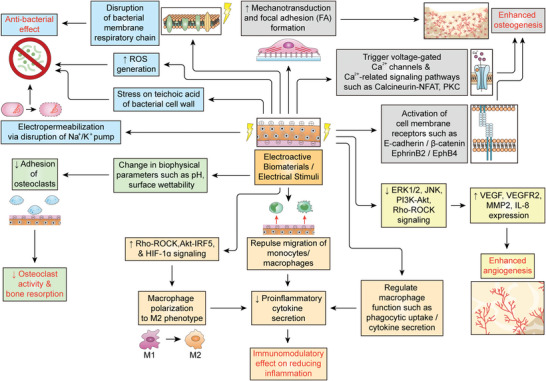
Putative mechanisms by which electroactive biomaterials or electrical stimuli promote bone regeneration under pathological conditions.

Electroactive scaffold materials can enhance osteogenic differentiation of mesenchymal stem cells, osteoblast precursors, and osteoblasts via three major signaling mechanisms (**Figure** **3**): i) focal adhesion (FA)‐associated mechanotransduction signaling axis, ii) intracellular Ca^2+^ and Ca^2+^‐activated signaling pathways, and iii) cell membrane‐bound receptor‐associated signaling pathways.^[^ ^107^ ^]^ Additionally, miscellaneous signaling pathways not associated with these major signaling mechanisms can also be activated.

Electroactive scaffolds have been reported to promote the clustering of FAs and the subsequent activation of focal adhesion kinase (FAK), which in turn trigger the mechanotransduction signaling axis to promote osteogenic differentiation via YAP/TAZ transcriptional co‐activation of key genes involved in osteogenesis.^[^ ^108^, ^109^ ^]^ The underlying mechanism is thought to involve a change in the conformation of adsorbed fibronectin via the surface electrical charge, which in turn enhances the binding and clustering of integrin receptors to promote focal adhesion complex formation.^[^ ^110^ ^]^

Another mechanism of electrical stimulation in osteogenesis involves the triggering of an intracellular influx of Ca^2+^ ions via modulation of voltage‐gated calcium channels or connexin 43.^[^ ^111^, ^112^, ^113^, ^114^ ^]^ Elevated cytosolic Ca^2+^ levels then promote osteogenic differentiation via the calcineurin/NFAT signaling pathway or the Protein Kinase C (PKC) signaling pathway.^[^ ^115^, ^116^, ^117^ ^]^

Additionally, electrical stimuli have been reported to trigger pro‐osteogenic signaling pathways via the activation of cell‐membrane bound receptors.^[^ ^118^, ^119^ ^]^ For example, Zhang et al.^[^ ^118^ ^]^ reported that the electrical stimuli‐induced changes in cell membrane potential caused the dissociation of *β*‐catenin from E‐Cadherin at the inner cell membrane surface, which in turn promoted osteogenesis via the activation of Wnt signaling. Luca et al.^[^ ^119^ ^]^ reported that a positively charged surface enhanced the interaction between membrane‐bound receptors EphrinB2 and EphB4 on adjacent cells, leading to enhanced osteogenesis.

Besides these three major signaling mechanisms, other pro‐osteogenic signaling pathways have also been reported to be triggered by electrical stimuli. These include ERK1/2,^[^ ^120^ ^]^ iNOS,^[^ ^121^ ^]^ and BMP/SMAD signaling pathways.^[^ ^122^ ^]^

### 4.2 Enhancement of Angiogenesis and Vascularization

Vascularization and angiogenesis play key roles in bone homeostasis, regeneration, and healing.^[^ ^123^ ^]^ Many studies have demonstrated that the application of exogeneous stimuli (e.g., electrical stimuli via electrodes) can promote vascularization and angiogenesis both in vivo and in vitro (Figure 3). However, no studies have directly demonstrated that electroactive materials can promote bone regeneration via the enhancement of angiogenesis. In the case of bone regeneration, the study of Fonseca et al.^[^ ^124^ ^]^ demonstrated that electrical stimuli could enhance bone defect healing by increasing VEGF expression, which resulted in enhanced vascularization at the injury site. Similarly, Sheikh et al.^[^ ^125^ ^]^ demonstrated the application of a high frequency electric field enhanced diabetic skin wound healing in a mouse model by enhancing capillary morphogenesis via increasing VEGF release and activation of the ERK/MAPK signaling pathway.

An in vitro study by Tzoneva et al.^[^ ^126^ ^]^ showed that application of a direct current on human umbilical vein endothelial cells (HUVEC) cultured on gelatin‐based hydrogels upregulated VEGF and MMP2 expressions, both of which play key roles in angiogenesis. Similarly, an in vitro study by Chen et al.^[^ ^127^ ^]^ reported the application of a small electric field on endothelial cells in a 3D culture enhanced VEGF and VEGFR2 expressions, and activated Akt, Erk1/2, and JNK signaling pathways. Zhao et al.^[^ ^128^ ^]^ reported the upregulated VEGFR receptor expression induced by the electric field was mediated through PI3K‐Akt and Rho‐ROCK signaling pathways, leading to actin cytoskeleton reorganization. A study by Bai et al.^[^ ^129^ ^]^ showed the application of an electric field on endothelial cells upregulated pro‐angiogenic factors such as IL‐8, VEGF121, and VEGF165.

Interestingly, electrical stimuli were also shown to induce angiogenic activity in non‐endothelial lineage cells. For example, Sauer et al.^[^ ^130^ ^]^ demonstrated the application of an electric field on embryonic stem cell‐derived embryoid bodies increased positive staining for the endothelial‐specific marker PECAM‐1, and upregulated VEGF and HIF‐1*α* expressions via activation of ERK1, 2, p38, and JNK signaling pathways. Ye et al.^[^ ^131^ ^]^ demonstrated that electrical stimulation of trophoblast cells upregulated VEGF expression via the AKT signaling pathway.

### 4.3 Inhibition of Osteoclastogenesis and Bone Resorption

To date, there had been very few studies on the effects of electrical stimuli and electroactive scaffolds on osteoclast function (Figure 3). In vivo studies on animal models of bone defect demonstrated repressed osteoclast formation upon implantation of hydroxyapatite‐based scaffolds polarized with a positive charge,^[^ ^132^, ^133^, ^134^ ^]^ but the underlying mechanisms were unclear. Nevertheless, an in vitro study by Bergara–Muguruza reported that electrical polarization enhanced the surface wettability of a synthetic carbonate‐substituted apatite scaffold and increased osteoclast resorption, but did not affect the early differentiation phase or the adherent morphology of the osteoclasts.^[^ ^135^ ^]^ Another in vitro study by Yao et al.^[^ ^136^ ^]^ reported that electrical stimulation could activate both osteoclasts and osteoblasts in a co‐culture system by inducing changes in the pH of the culture medium.

### 4.4 Immunomodulatory Effects

As previously discussed in Section 3, many diseases can lead to bone degeneration and loss via inflammation, which involve the recruitment of macrophages and other inflammatory cells to the bone defect site.^[^ ^137^ ^]^ Electroactive scaffolds and electrical stimuli have been demonstrated to exert immunomodulatory effects (Figure 3), which can mitigate inflammation to promote bone healing and regeneration. Studies have shown that monocytes and macrophages tend to migrate away from electrical stimuli.^[^ ^138^, ^139^ ^]^ Electroactive materials could therefore exploit this phenomenon to exert an anti‐inflammatory effect by directly repelling inflammatory cells, leading to reduced secretion of proinflammatory cytokines at the bone defect site. Electrical stimulation has also been shown to significantly promote macrophage phagocytic uptake, selectively regulate cytokine production,^[^ ^140^ ^]^ and facilitate the transition of macrophages from the proinflammatory M1 to the pro‐healing M2 phenotype.^[^ ^141^, ^142^, ^143^ ^]^

Dai et al.^[^ ^141^ ^]^ reported that hyperglycemia associated with diabetes resulted in the polarization of macrophages toward the proinflammatory M1 phenotype, which in turn hindered bone defect healing. This was attenuated by covering the bone defect site with a BTO/P(VDF‐TrFE) ferroelectric nanocomposite membrane, which restored the physiological electrical microenvironment of the bone under diabetic conditions. This induced M1 macrophages to the M2 phenotype concomitant with the reduction in IL‐6 secretion, which enhanced the osteoimmunomodulatory environment for bone defect healing and regeneration under diabetic conditions. Further investigation revealed the immunomodulatory effects of the electroactive nanocomposite membrane on macrophages was mediated via the AKT2‐IRF5/HIF‐1*α* signaling pathway.

Li et al.^[^ ^142^ ^]^ reported a polydopamine‐mediated graphene oxide (PGO) and hydroxyapatite nanoparticle‐incorporated conductive alginate/gelatin (AG) scaffold that enhanced periodontal bone regeneration by mitigating the diabetic inflammatory microenvironment. Similar to the study by Dai et al.,^[^ ^141^ ^]^ this electroactive scaffold promoted the transition of M1 macrophages to the M2 phenotype concomitant with the reduction of proinflammatory cytokine levels. The mechanistic study further implicated the involvement of the RhoA/ROCK signaling pathway.

In a related study by Jiang et al.,^[^ ^143^ ^]^ spherical mannose‐decorated globular lysine dendrimers (MGLDs) with a positive surface charge induced mouse bone marrow‐derived macrophages to acquire a pro‐healing M2 phenotype, concomitant with the decreased secretion of proinflammatory cytokines such as TNF‐*α*, IL‐1*β*, and IL‐6. Although this study did not use a bone defect model, it did demonstrate that the electroactive material could enhance the healing of full‐thickness cutaneous defects in type 2 diabetic mice via M2 macrophage polarization.

### 4.5 Anti‐Bacterial Effects

Electroactive materials with anti‐bacterial effects (Figure 3) would be particularly advantageous for promoting bone tissue healing and regeneration under disease conditions involving pathogenic bacterial infections such as periodontitis (Section 3.3) and osteomyelitis (Section 3.6). To date, numerous electroactive biomaterials with anti‐bacterial properties have been developed that have distinct advantages over scaffold biomaterials with incorporated bactericides and/or controlled release of anti‐bacterial agents.^[^ ^144^ ^]^ For example, some electroactive materials have anti‐bacterial properties that can prevent infection by antibiotic‐resistant bacteria and have longer‐lasting effects than anti‐microbial peptides with limited active half‐life in vivo, as well as being more cost‐effective.^[^ ^144^ ^]^ Moreover, electroactive biomaterials could be designed to be more biocompatible compared to bactericidal agents such as CuO and ZnO nanoparticles that are cytotoxic at high concentration.^[^ ^144^ ^]^

A major mechanism through which electroactive materials exert their bactericidal effect is by increasing cell membrane permeability, which is termed electropermeabilization or irreversible electroporation.^[^ ^145^ ^]^ This arises from the disruption of Na^+^/K^+^ pump activity by the electroactive material, leading to hyperpolarization or depolarization of the bacterial cell membrane.^[^ ^146^ ^]^ In addition, electrical stimuli can induce stress within the teichoic acids of the bacterial cell wall leading to pore formation.^[^ ^147^ ^]^ Electroactive materials could exploit other bactericidal mechanisms including the generation of ROS,^[^ ^148^, ^149^ ^]^ and disruption of the respiratory chain on the bacterial membrane.^[^ ^150^ ^]^

## 5 Electroactive Biomaterials for Bone Regeneration

### 5.1 Piezoelectric Biomaterials

Piezoelectric biomaterials are capable of generating an electrical signal in response to a mechanical load or deformation of the material.^[^ ^151^ ^]^ Moreover, piezoelectric biomaterials can also produce an opposite effect (reverse piezoelectric effect), whereby the application of an external electrical stimulus results in a mechanical force or deformation of the material.^[^ ^151^ ^]^ Piezoelectric materials are noncentrosymmetric in nature, with the mechanisms by which the piezoelectricity is generated differing depending on the use of organic and inorganic materials.^[^ ^151^ ^]^ In the case of organic materials, the piezoelectric mechanism involves the reorientation of molecular dipoles within the bulk polymer structure (polarization) under mechanical deformation, leading to the formation of a net dipole moment that in turn generates an electrical signal.^[^ ^151^ ^]^ In the case of inorganic materials, the piezoelectric mechanism involves the displacement and subsequent shift in the balance of ions within the crystalline structure under mechanical stress, leading to the creation of a dipole moment that generates an electrical signal.^[^ ^151^ ^]^ In both cases, the reorientation of positive and negative charges within the material induced by the mechanical force or deformation leads to the generation of a microcurrent. Because piezoelectric biomaterials can generate electrical stimuli through natural bodily movement and routine physical activity without the need for an external power source, they have attracted much interest for their potential application as implantable scaffolds in bone tissue engineering.^[^ ^10^, ^152^ ^]^ On the other hand, the use of in vivo implanted scaffold materials that can generate piezoelectric signals via external stimuli such as ultrasound or electromagnetic fields have also been reported to enhance bone defect healing in situ.^[^ ^153^, ^154^ ^]^ Piezoelectric biomaterials commonly utilized for bone regeneration can be broadly divided into three categories: i) synthetic piezoelectric polymers, ii) synthetic piezoceramics, and iii) naturally occurring piezoelectric materials in either polymeric or ceramic form. Additionally, various piezocomposite materials that combine and synergize the advantageous properties of two or more synthetic or natural piezoelectric biomaterials have also been studied.^[^ ^10^, ^152^ ^]^ Many excellent reviews on piezoelectric biomaterials have already been published within the scientific literature, so only brief descriptions will be given here.

Synthetic piezoelectric polymers are characterized by their high flexibility and low stiffness, which can be precisely controlled during fabrication depending on the specific requirements of the tissue/organ of the implant site.^[^ ^155^ ^]^ Another major advantage of piezoelectric polymers is their amenability to be fabricated into diverse structures including films, hydrogels, microspheres, and nanofibers by different fabrication techniques such as spin coating, electrospinning, and template methods.^[^ ^155^ ^]^ The most common synthetic piezoelectric polymers used in bone tissue engineering are poly(vinylidene fluoride) (PVDF) and its co‐polymers such as poly(vinylidene fluoride‐co‐tetrafluoroethylene) (PVDF–TrFE) due to their good piezoelectric response and excellent mechanical properties.^[^ ^156^, ^157^ ^]^ However, these materials are not biodegradable, which may pose a clinical challenge.^[^ ^156^, ^157^ ^]^ For example, they can be utilized as covers (membranes or films) for bone defects, but subsequent surgery is often required for their removal after bone defect healing. On the other hand, when used as filling materials for bone defects, it is virtually impossible to remove these materials as they become integrated into newly formed bone tissues, hence, their permanent presence in vivo could lead to detrimental effects such as provoking inflammatory reactions.^[^ ^156^, ^157^ ^]^ To this end, biodegradable and bioabsorbable piezoelectric polyesters such as Poly‐L‐lactic acid (PLLA),^[^ ^158^ ^]^ Polyhydroxybutyrate (PHB),^[^ ^159^ ^]^ and polyhydroxybutyrate‐co‐valerate (PHBV),^[^ ^160^ ^]^ are often preferred for clinical applications in bone defect healing and bone tissue engineering, even though they may have less favorable mechanical properties compared to PVDF and PVDF–TrFE.

Synthetic piezoceramic materials have similar mechanical properties to natural bone tissue, such as high hardness and friction coefficients, although they are usually quite brittle. Therefore, piezoceramic materials are often combined with other polymeric biomaterials such as piezoelectric polymers or conventional biodegradable polymers to form piezocomposite materials for clinical applications in bone regeneration.^[^ ^161^, ^162^ ^]^ Alternatively, piezoceramics can also be applied as powders or nanoparticles. The most common piezoceramic materials used in bone regeneration include barium titanium oxide (BaTiO_3_, BTO),^[^ ^163^ ^]^ potassium sodium niobate (KNN),^[^ ^164^ ^]^ and bioactive glass (BG).^[^ ^165^ ^]^ Although zinc is a key trace element and vital to bone tissue development, ZnO is less commonly used as a piezoceramic material, as it is cytotoxic.^[^ ^166^, ^167^ ^]^ Nevertheless, ZnO has demonstrated anti‐bacterial properties, which would be advantageous for bone defect healing.

Finally, some naturally occurring piezoelectric biomaterials (polymeric or ceramic) with good biodegradability, excellent biocompatibility, and negligible cytotoxicity,^[^ ^168^ ^]^ have been used in clinical applications for bone regeneration, such as collagen and hydroxyapatite,^[^ ^169^, ^170^ ^]^ which are also natural components of bone. Indeed, deproteinized bovine bone extracellular matrix consisting mainly of hydroxyapatite is widely used in orthopedic surgery.^[^ ^171^ ^]^ Other naturally occurring piezoelectric polymers commonly used in bone regeneration include cellulose,^[^ ^172^ ^]^ chitin,^[^ ^173^ ^]^ and chitosan.^[^ ^174^ ^]^

### 5.2 Electroconductive Biomaterials

As bone is an electroactive and electroresponsive tissue (Section 2), its regeneration can be promoted by the implantation of electroconductive biomaterials that enable electron transport at the cell‐substrate interface, which in turn facilitate cell‐substrate interaction, cross talk, and intercellular communication.^[^ ^175^, ^176^ ^]^ Indeed, some studies have demonstrated that electroconductive biomaterials can enhance the osteogenesis of adult stem cells and osteoprogenitors without any exogenous electrical stimuli.^[^ ^177^, ^178^ ^]^ Electroconductive biomaterials used in bone regeneration and tissue engineering can be broadly divided into three categories: i) carbon‐based biomaterials, ii) metal/metal oxides, and iii) conductive polymers. Numerous excellent reviews on electroconductive biomaterials have already been published, so only brief descriptions will be given here.

The most common carbon‐based electroconductive biomaterials used for bone regeneration are graphene,^[^ ^179^ ^]^ carbon nanotubes,^[^ ^180^ ^]^ and carbon nanofibers.^[^ ^181^ ^]^ In addition, carbon dots (C‐dots), fullerenes, and nanodiamonds have also been increasingly used in clinical orthopedic applications.^[^ ^182^, ^183^ ^]^ Although carbon‐based electroconductive biomaterials consist mainly of carbon atoms, the different atomic arrangements in the form of various carbon allotropes confer vastly different mechanical properties and surface chemistries.^[^ ^179^, ^180^, ^181^, ^182^, ^183^ ^]^ Besides their favorable mechanical properties and high electrical conductivity, these carbon‐based biomaterials have large specific surface area that is amenable to the attachment of a diverse array of bioactive functional groups, which can also facilitate the loading and controlled release of small molecules and peptide‐based drugs, making them particularly advantageous for clinical orthopedic applications.^[^ ^179^, ^180^, ^181^, ^182^, ^183^ ^]^ Moreover, the fabrication of composite biomaterials with non‐conductive biopolymers such as collagen and chitosan often use carbon‐based biomaterials as fillers to confer electrical conductivity and also provide mechanical strength required for load‐bearing bone repair.^[^ ^184^ ^]^

Metallic alloys composed of steel, titanium, and magnesium have high mechanical strength and are inherently electroconductive, which makes them ideal as orthopedic implant materials for load‐bearing bone repair.^[^ ^185^ ^]^ Some of the new generation of metallic alloy‐based implants are designed to be porous, which enhances stress conduction, facilitates the loading of therapeutic drugs, and allows vascularization within the implants.^[^ ^185^ ^]^ More recently, highly conductive gold (Au)‐based micro and nanomaterials have been found to be useful as reinforcing materials, in composite scaffold implants for bone regeneration, as they can provide both mechanical stiffness and flexibility, as well as being non‐reactive with good biocompatibility.^[^ ^186^ ^]^ Moreover, Au nanomaterials are also amenable to facile surface modification via gold‐thiol chemistry, which can facilitate drug loading.^[^ ^186^ ^]^ Other metal/metal oxide nanomaterials based on Ag, B, Cu, Mg, Pt, Sr, Ti, Zn, and MoS_2_ have also been employed in bone repair and tissue engineering.^[^ ^187^ ^]^

The most common electroconductive polymers used in bone regeneration include Polypyrrole (PPy),^[^ ^188^ ^]^ polyaniline (PANi),^[^ ^189^ ^]^ and poly(3,4‐ethylenedioxythiophene) (PEDOT).^[^ ^190^ ^]^ Although these materials have been shown to be biocompatible and conducive to osteogenesis,^[^ ^188^, ^189^, ^190^ ^]^ they have poor processibility and are insoluble or poorly soluble in most solvents, while their conjugated molecular backbone makes them mechanically rigid and brittle, and non‐degradable. To overcome their poor processibility, electroconductive polymers are usually deposited or combined with other materials, which allows shaping and molding for implantation or cell culture.^[^ ^191^, ^192^ ^]^ Nevertheless, the non‐biodegradability of these polymers in vivo may pose a safety hazard, particularly after other supporting materials have degraded. One solution is to use these materials as conductive oligomers within the composite scaffolds.^[^ ^191^, ^192^ ^]^

### 5.3 Electrostimulation Scaffolds with Implantable Energy Harvesters

The human body contains various forms of potential energy that can be harnessed by implantable energy harvesters (IEH) to generate electrical stimuli for bone regeneration.^[^ ^193^ ^]^ These sources of energy include mechanical motion generated by the musculoskeletal system during daily physical activities such as walking, blood flow, heartbeat, and respiration, which can be harvested by piezoelectric nanogenerators (PENGs),^[^ ^194^ ^]^ triboelectric nanogenerators (TENGs),^[^ ^195^ ^]^ and mass imbalance oscillation generators (MIOG).^[^ ^196^ ^]^ Additionally, electrochemical energy within the human body can also be harnessed to provide electrical stimuli through the use of enzymatic biofuel cells (EBFCs) and endocochlear potential (EP) collectors.^[^ ^197^, ^198^ ^]^ Less commonly used harvesters include photovoltaic cells (PVC) and pyroelectric nanogenerators (PYENGs) that harvest light and heat energy from the environment.^[^ ^199^, ^200^ ^]^ The use of IEH‐based electrostimulation scaffolds for bone regeneration and other tissue engineering applications merit their own comprehensive review, which is beyond the scope of this article.

### 5.4 Electroresponsive Biomaterials

Some new‐generation electroactive biomaterials are also electroresponsive, which means they are capable of changing their biochemical, biophysical, or microenvironmental properties in response to external electrical stimuli. For example, in electroresponsive drug delivery systems based on electro‐redox reactions of conducting polymers such as polypyrrole, the positive charge is lost upon electro‐reduction of its oxidized state to enable drug release.^[^ ^201^ ^]^ Additionally, electrical stimuli can be used to control drug release from pH‐responsive scaffolds by electro‐induced pH changes.^[^ ^202^ ^]^ Besides drug release, electroresponsive biomaterials can also be used to effect changes in cell functions such as adhesion, differentiation, and proliferation in response to electrical stimuli. For example, the RGD tripeptide sequence (Arg‐Gly‐Asp), which is widely expressed by ECM proteins and regulates integrin‐mediated cell adhesion, can change conformation upon electrical stimulation.^[^ ^203^ ^]^ The extent of the conformational changes can be altered by adjusting the exposure of RGD sequences to electrical stimuli. In this way, electroresponsive biomaterials incorporating RGD peptides can be used to regulate cell adhesion and proliferation.^[^ ^204^, ^205^ ^]^ Cell function can also be regulated by electroresponsive biomaterials through motion or mechanical force. For example polyelectrolyte hydrogels that can undergo swelling and deswelling in response to pulsatile electrostimulation can mechanically stimulate cells.^[^ ^206^, ^207^ ^]^

## 6 Electroactive Scaffolds Can Facilitate Bone Regeneration under Various Disease Conditions

### 6.1 Diabetes

To date, only a few studies have utilized electroactive biomaterials to promote the healing of bone and other tissues under diabetic conditions.^[^ ^141^, ^142^, ^143^ ^]^ These studies demonstrated that electroactive biomaterials were able to facilitate bone regeneration under diabetic conditions by suppressing inflammation and osteoclastogenesis,^[^ ^141^, ^142^, ^143^, ^208^ ^]^ and enhancing osteogenesis.^[^ ^142^ ^]^

Dai et al.^[^ ^141^ ^]^ showed the inflammatory action of macrophages was enhanced by hyperglycemia associated with type II diabetes, which in turn hindered bone defect healing in a rat model. They subsequently showed the implantation of a polarized BaTiO_3_/P(VDF‐TrFE) nanocomposite membrane suppressed macrophage‐mediated inflammation and enhanced bone defect healing.^[^ ^141^ ^]^ This electroactive biomaterial promoted macrophage phenotype transition from M1 to M2 via suppressing the expression of AKT2 and IRF5 within the PI3K‐AKT signaling pathway, which induced a favorable osteoimmunomodulatory environment to enhance bone defect healing.^[^ ^141^ ^]^ Similarly, Li et al.^[^ ^142^ ^]^ developed an electroactive polydopamine‐mediated graphene oxide (PGO) and hydroxyapatite nanoparticle (PHA)‐incorporated conductive alginate/gelatin (AG) scaffold, which accelerated periodontal bone regeneration in a diabetic rat model. This scaffold promoted the polarization of macrophages to the pro‐healing M2 phenotype via modulating glycolytic and RhoA/ROCK pathways. The M2 macrophages were shown to contribute to an osteoinductive environment via the secretion of osteogenesis‐related cytokines, which in turn enhanced periodontal bone defect healing.^[^ ^142^ ^]^ Similar immunomodulatory effects were also reported in the study by Jiang et al.,^[^ ^143^ ^]^ which showed positively charged mannose‐decorated globular lysine dendrimers (MGLDs) could enhance the shift to the M2 phenotype in mouse bone marrow‐derived macrophages. This was attributed to their elongated shape and significant clustering of mannose receptors (MR) on the cell surface, besides decreased secretion of proinflammatory cytokines. Although this in vivo study did not examine bone defect repair, it demonstrated that MGLDs could enhance cutaneous wound healing in a diabetic mouse model.^[^ ^143^ ^]^

Besides immunomodulatory effects on macrophages, electroactive materials can promote bone defect healing through enhancing osteogenesis. A study by Li et al.^[^ ^142^ ^]^ showed an electroconductive polydopamine‐mediated graphene oxide (PGO) and hydroxyapatite nanoparticle (PHA)‐incorporated conductive alginate/gelatin (AG) scaffold could promote periodontal bone regeneration in a diabetic rat model by activating Ca^2+^ channels via endogenous electrical stimuli. Another possible mechanism by which electroactive materials can promote bone defect healing under diabetic conditions is through suppressing osteoclastogenesis. This was demonstrated in an in vitro study by Rother et al.^[^ ^209^ ^]^ using a negatively charged collagen‐glycosaminoglycan surface coating, although these results need to be validated in an in vivo animal study.

### 6.2 Osteoporosis

To date, there have been no studies that have directly utilized electroactive biomaterials to facilitate bone defect healing under osteoporosis conditions in either animal or human models. Nevertheless, some studies strongly suggest that electroactive scaffolds could exert beneficial therapeutic effects. For example, a theoretical *in silico* simulation of bone remodeling by Bansod et al.^[^ ^210^ ^]^ predicted that electrical stimuli can increase bone mineral density, which would be of therapeutic benefit to osteoporosis patients. Lirani‐Galvão et al.^[^ ^211^ ^]^ demonstrated that whole‐body stimulation with a low‐intensity pulsed electrical field could enhance bone mineral density in an ovariectomized rat model of osteoporosis. Similar results were obtained by Manjhi et al.,^[^ ^212^ ^]^ who demonstrated that a capacitively coupled pulsed electric field (CCPEF) could prevent bone loss in an ovariectomized rat model of osteoporosis. Histopathological analyses revealed that localized treatment with CCPEF also augmented and restored the bone marrow cell population, increased collagen fiber density, and improved the microstructural composition and compactness of the treated bone tissues.^[^ ^213^ ^]^ Additionally, the immunohistological analysis showed increased alkaline phosphatase (ALP) activity after electrostimulation, which enhanced osteoblast differentiation.^[^ ^213^ ^]^ The use of charged PLGA nanoparticles was reported to enhance delivery of estradiol (E2) compared to uncharged nanoparticles, but the observed therapeutic effects on osteoporosis were attributed to the enhanced E2 delivery rather than the electrical stimulation from the charged nanoparticles.^[^ ^214^ ^]^

### 6.3 Periodontitis

No studies have directly utilized electroactive biomaterials to promote periodontal bone regeneration under periodontitis conditions. Nevertheless, indirect scientific evidence from other studies suggest that electroactive biomaterials could show therapeutic benefit for bone defect healing in periodontitis. Considering periodontitis is a chronic inflammatory disease caused by oral pathogens,^[^ ^47^, ^48^ ^]^ electroactive materials with anti‐inflammatory activity could promote the transition of macrophage from the proinflammatory M1 phenotype to the pro‐healing M2 phenotype to enhance bone regeneration via a similar mechanism to bone defect healing under diabetic conditions.^[^ ^141^, ^142^, ^143^ ^]^ An interesting study by Chakraborty et al.^[^ ^215^ ^]^ demonstrated the presence of LPS or bacterial cells could lead to significant changes in the surface electrical potential of macrophage, which correlates with the transition of M1/M2 phenotype. Hence, it is possible that changes in the surface electrical potential of macrophages could affect the immunomodulatory activity of electroactive biomaterials, although this needs further validation.

A study by Nohara et al.^[^ ^216^ ^]^ demonstrated that implantation of an electrically polarized *β*‐tricalcium phosphate scaffold could enhance bone defect healing in canine maxilla, although this was not a periodontitis model. To date, a number of studies have demonstrated the beneficial effects of direct electrical stimulation on alveolar bone regeneration without the use of electroactive biomaterials.^[^ ^217^, ^218^, ^219^ ^]^ Kaynak et al.^[^ ^217^ ^]^ showed the application of a capacitively coupled electrical field (CCEF) could enhance alveolar bone defect repair in a beagle dog model. Similarly, Bins‐Ely et al.^[^ ^218^ ^]^ demonstrated that direct electrical stimulation could promote bone formation around titanium dental implants in a beagle dog model. Cosoli et al.^[^ ^219^ ^]^ reported the application of a radio frequency current could mitigate inflammation (peri‐implantitis) around dental implants. Lastly, Kim et al.^[^ ^220^ ^]^ reported that direct electrical stimulation via an implantable liquid crystal polymer‐based electrode promoted substantial bone defect healing in rabbit mandibles, but not in alveolar bone.

Other studies have demonstrated that electrical stimuli can exert antimicrobial effects, which would be beneficial for the treatment of periodontitis. Obermeier et al.^[^ ^221^ ^]^ reported the application of a low‐frequency electric field could inhibit the growth of *Staphylococcus aureus*. Schmidt‐Malan et al.^[^ ^222^ ^]^ also showed the application of direct electrical current on bones infected with *Staphylococcus epidermidis* could mitigate inflammation and enhance bone defect healing.

### 6.4 Osteoarthritis and Rheumatoid Arthritis

To date, there have been no studies on either direct electrical stimulation or implantation of electroactive materials to promote subchondral bone regeneration in OA and RA, whereas the overwhelming majority of studies on OA and RA have focused on the regeneration of articular cartilage rather than subchondral bone. It should be noted that the degeneration of subchondral bone under OA and RA pathological conditions are mainly due to inflammation that promotes increased osteoclastogenesis (Sections 3.4 and 3.5). As discussed earlier, electroactive biomaterials have been demonstrated to have both anti‐osteoclastogenic and anti‐inflammatory effects (Sections 4.3 and 4.4, respectively), which could be exploited to promote subchondral bone regeneration under OA and RA disease conditions.

### 6.5 Osteomyelitis and Metastatic Osteolysis

Currently, no studies that have investigated the effects of either direct electrical stimulation or implantation of electroactive biomaterials on promoting bone regeneration under osteomyelitis and metastatic osteolysis conditions. Nevertheless, available scientific evidence strongly suggest that electroactive biomaterials could exert beneficial therapeutic effects on osteomyelitis and metastatic osteolysis. As discussed earlier, osteomyelitis is characterized by inflammation due to bacterial infection, which in turn increases osteoclastogenesis and bone resorption (Section 3.6). Electroactive biomaterials that demonstrate anti‐osteoclastogenic (Section 4.3), anti‐inflammatory (Section 4.4), and anti‐bacterial (Section 4.5) effects could show therapeutic benefits for osteomyelitis. Similarly, the increased osteoclastogenesis and bone resorption characterized in metastatic osteolysis (Section 3.7) could be mitigated by electroactive biomaterials with anti‐osteoclastogenic activity (Section 4.3).

## 7 Conclusions and Future Perspectives

The rapidly aging worldwide population and the increasing sedentary lifestyles and unhealthy diets associated with urban environments have fueled the rising incidence of degenerative bone conditions associated with various diseases such as type II diabetes, osteoporosis, periodontitis, osteoarthritis, rheumatoid arthritis, osteomyelitis, and metastatic osteolysis. Given that bone is an electroactive and electroresponsive tissue, and that bone degeneration under various pathological conditions is associated with significant changes in its bioelectric properties, it is plausible that restoring the natural electrophysiological microenvironment could promote bone healing and regeneration. This may be achieved through the use of biomimetic electroactive materials that exert beneficial therapeutic effects via enhancement of osteogenesis and angiogenesis, suppression of inflammation and osteoclastogenesis, and protection against bacterial infection (**Table** **3**). Of particular interest are the new generation of novel electroactive biomaterials (**Table** **4**) that utilize implantable energy harvesters (Section 5.3) or electroresponsive materials that can alter their properties in response to the microenvironment (Section 5.4). Also of interest are the design of piezoelectric biomaterials that may be combined with the application of external ultrasound and magnetic fields to generate stronger, more stable and longer‐lasting electrical stimuli in situ after implantation. Additionally, this will also allow the magnitude and frequency of electrical stimuli to be fine‐tuned on demand, to meet the specific clinical needs of bone defect repair under different pathological conditions, thereby maximizing the therapeutic efficacy of electroactive biomaterials. To date, the overwhelming majority of studies on bone tissue engineering and bone graft materials have focused mainly on fractures and defects arising from traumatic injuries, whereas much less attention been paid to degenerative bone conditions associated with various disease pathologies. It is hoped that this review will encourage more research efforts on developing innovative biomaterials for augmenting defective bone regeneration under various pathological conditions.

**Table 3:** Mechanisms by which electroactive scaffolds or electrical stimuli promote bone healing and regeneration

Enhancement of osteogenesis	Focal adhesion (FA) associated mechanotransduction signaling pathway	Raic et al.^[^ ^108^ ^]^ Shen et al.^[^ ^109^ ^]^ Ribeiro et al.^[^ ^110^ ^]^
	Voltage‐gated Ca^2+^ channels	Bagne et al.^[^ ^111^ ^]^ Zhuang et al.^[^ ^112^ ^]^ Brighton et al.^[^ ^113^ ^]^
	Connexin 43 mediated influx of Ca^2+^	Park et al.^[^ ^114^ ^]^
	Calcineurin/NFAT signaling	Winslow et al.^[^ ^115^ ^]^ Wang et al.^[^ ^116^ ^]^
	Protein Kinase C (PKC) signaling	Shen et al.^[^ ^109^ ^]^ Liu et al.^[^ ^117^ ^]^
Enhancement of angiogenesis/vascularization	Secretion of VEGF and other pro‐angiogenic cytokines	Fonseca et al.^[^ ^124^ ^]^ Tzoneva et al.^[^ ^126^ ^]^ Bai et al.^[^ ^129^ ^]^
	ERK/MAPK signaling	Sheikh et al.^[^ ^125^ ^]^
	Akt – ERK1/2 – JNK signaling axis	Chen et al.^[^ ^127^ ^]^
	PI3K – Akt/Rho‐ROCK signaling axis	Zhao et al.^[^ ^128^ ^]^
Inhibition of osteoclastogenesis and bone resorption	Mechanisms unclear	
Immunomodulatory effects	Monocytes and macrophages migrate away from electrical stimuli	Leppik et al.^[^ ^138^ ^]^ Kearns & Thompson^[^ ^139^ ^]^
	Enhancement of macrophage phagocytic uptake	Hoare et al.^[^ ^140^ ^]^
	Promote transition of macrophages from pro‐inflammatory M1 to pro‐healing M2 phenotype	Dai et al.^[^ ^141^ ^]^ Li et al.^[^ ^142^ ^]^ Jiang et al.^[^ ^143^ ^]^
Anti‐bacterial effects	Electropermeabilization via disruption of Na^+^/K^+^ pump	Valic et al.^[^ ^146^ ^]^
	Electropermeabilization via stress induction on teichoic acid of bacterial cell wall	Rauch & Leigh^[^ ^147^ ^]^
	ROS generation	Jeong et al.^[^ ^148^ ^]^ Feng et al.^[^ ^149^ ^]^
	Disruption of bacteria respiratory chain	Wang et al.^[^ ^150^ ^]^

**Table 4:** Electroactive biomaterials that can potentially be utilized for promoting bone healing and regeneration under various disease conditions

Electroactive biomaterials	Category/Type	Advantages	Disadvantages	Key references
Piezoelectric biomaterials	Synthetic piezopolymers	High flexibility and low stiffness	Low biodegradability	Ribeiro et al.^[^ ^155^ ^]^ Kalimuldina et al.^[^ ^156^ ^]^ Rufato et al.^[^ ^157^ ^]^ Capuana et al.^[^ ^158^ ^]^ Williams^[^ ^159^ ^]^ Goonoo et al.^[^ ^160^ ^]^
	Synthetic piezoceramics	Similar mechanical properties to natural bone tissue, such as high hardness and friction coefficients	Very brittle	Li et al.^[^ ^163^ ^]^ Chen et al.^[^ ^164^ ^]^ El‐Rashidy et al.^[^ ^165^ ^]^ Felice et al.^[^ ^166^ ^]^ Ziglari et al.^[^ ^167^ ^]^
	Naturally‐occurring piezoelectric materials in polymeric or ceramic form	Good biodegradability, excellent biocompatibility, and negligible cytotoxicity	Higher inter‐batch variability than synthetic biomaterials	Shin et al.^[^ ^168^ ^]^ Rico‐Llanos et al.^[^ ^169^ ^]^ Arcos & Vallet‐Regí^[^ ^170^ ^]^ Baldini et al.^[^ ^171^ ^]^ Osorio et al.^[^ ^172^ ^]^ Jayakumar et al.^[^ ^173^ ^]^ Aguilar et al.^[^ ^174^ ^]^
Electroconductive biomaterial	Carbon‐based biomaterials	Good mechanical properties High electrical conductivity Large specific surface area for loading of bioactive factors	Low biodegradability Some degree of cytotoxicity	Shadjou et al.^[^ ^179^ ^]^ Tanaka et al.^[^ ^180^ ^]^ Aoki et al.^[^ ^181^ ^]^ Peng et al.^[^ ^182^ ^]^ Liu et al.^[^ ^183^ ^]^
	Metal/metal oxides	Good mechanical properties High electrical conductivity	Low biodegradability Some degree of cytotoxicity	Wang et al.^[^ ^185^ ^]^ Li et al.^[^ ^186^ ^]^ Wang et al.^[^ ^187^ ^]^
	Conductive polymers	High electrical conductivity	Rigid and brittle Low biodegradability	Liang and Goh.^[^ ^188^ ^]^ Rajzer et al.^[^ ^189^ ^]^ Guex et al.^[^ ^190^ ^]^
Electrostimulation scaffolds/devices with implantable energy harvestors (IEH)	Piezoelectric nanogenerators (PENGs)	Generate electrical stimuli without an external power source by harvesting energy from the human body	Most of these technologies not yet mature, and face various challenges such as poor biodegradability, cytotoxicity and insufficient miniaturization	Kao et al.^[^ ^194^ ^]^
	Triboelectric nanogenerators (TENGs)			Li et al.^[^ ^195^ ^]^
	Mass imbalance oscillation generators (MIOG)			Zurbuchen et al.^[^ ^196^ ^]^
	Enzymatic biofuel cells (EBFCs)			Haque et al.^[^ ^197^ ^]^
	Endocochlear potential (EP) collectors			Mercier et al.^[^ ^198^ ^]^
	Photovoltaic cells (PVC)			Long et al.^[^ ^199^ ^]^
	Pyroelectric nanogenerators (PYENGs)			Ryu & Kim^[^ ^200^ ^]^
Electroresponsive biomaterials	Drug‐delivery	Enable precisely‐timed drug release via electrical stimuli	Requires direct electrical stimulation, which maybe difficult to apply to implants embedded deep within the human body	Sirivisoot et al.^[^ ^201^ ^]^ Kiaee et al.^[^ ^202^ ^]^
	Modulation of cell function – adhesion, proliferation and differentiation	Enable precise control of cellular function via electrical stimuli		Lashkor et al.^[^ ^203^ ^]^ Zhang et al.^[^ ^204^ ^]^ Tang et al.^[^ ^205^ ^]^
	Mechanostimulation	Enable precisely‐timed mechanostimulation via electrical stimuli		Shang et al.^[^ ^206^ ^]^ Rahimi et al.^[^ ^207^ ^]^

To achieve optimal bone repair and regeneration, electrical stimuli by itself would probably be insufficient. Perhaps the electroactive properties of biomaterials can be combined together with other biomechanical cues such as surface topography patterns, to synergistically enhance bone regeneration.^[^ ^223^, ^224^ ^]^ Additionally, the electroactive properties of biomaterials can also be exploited for the loading and delivery of therapeutic bioactive factors.^[^ ^225^ ^]^ For example, electroresponsive nano‐biomaterials can be potentially employed to deliver drugs or therapeutic transgenes to further enhance bone tissue regeneration.^[^ ^226^, ^227^ ^]^

Besides bone tissue engineering and orthopedic surgery, the development of biomimetic electroactive scaffolds can also benefit the repair and regeneration of other tissues and organs under adverse pathological conditions, given the various commonalities in the beneficial effects of electrical stimuli on tissue regeneration, as outlined in Section 4. (i.e., enhancement of angiogenesis, anti‐inflammatory, and anti‐bacterial properties). Indeed, electroactive scaffolds have also been reported to promote the regeneration of neural,^[^ ^228^ ^]^ cardiac,^[^ ^229^ ^]^ and skeletal muscle tissues.^[^ ^230^ ^]^

## Conflict of Interest

The authors declare no conflict of interest.
